# Novel receptor tyrosine kinase targeted combination therapies for imatinib-resistant gastrointestinal stromal tumors (GIST)

**DOI:** 10.18632/oncotarget.3021

**Published:** 2014-12-10

**Authors:** Daruka Mahadevan, Noah Theiss, Carla Morales, Amy E. Stejskal, Laurence S. Cooke, Min Zhu, Drew Kurtzman, Rachel Swart, Evan Ong, Wenqing Qi

**Affiliations:** ^1^ West Cancer Center/University of Tennessee Health Science Center (UTHSC), Memphis, TN; ^2^ University of Arizona Cancer Center, Tucson, AZ

**Keywords:** GIST, c-Kit, HER-1, imatinib, amuvatinib, erlotinib

## Abstract

Background: c-Kit/α-PDGFR targeted therapies are effective for gastrointestinal stromal tumors (GIST), but, >50% develop drug resistance.

Methods: RTK expression (c-Kit, c-Met, AXL, HER-1, HER-2, IGF-1R) in pre-/post-imatinib (IM) GIST patient samples (n=16) and 4 GIST cell lines were examined for RTK inhibitor activity. GIST-882 cells were cultured in IM every other day, cells collected (1 week to 6 months) and analyzed by qRT-PCR and Western blotting.

Results: Immunohistochemistry pre-/post-IM demonstrated continued expression of c-Kit and HER1, while a subset expressed IGF-1R, c-Met and AXL. In GIST cells (GIST-882, GIST430/654, GIST48) c-Kit, HER1 and c-Met are co-expressed. Acute IM over-express c-Kit while chronic IM, lose c-Kit and HER-1 in GIST882 cells. GIST882 and GIST430/654 cells have an IC_50_ 0.077 and 0.59 μM to IM respectively. GIST48 have an IC_50_ 0.66 μM to IM, 0.91 μM to amuvatinib [AMU] and 0.67 μM to erlotinib (Erl). Synergistic combinations: GIST882, AMU + Erl (CI 0.20); IM + AMU (CI 0.50), GIST430/654, IM + afatinib (CI 0.39); IM + AMU (CI 0.42), GIST48, IM + afatinib (CI 0.03); IM + AMU (CI 0.04); AMU + afatinib (CI 0.36); IM + Erl (CI 0.63).

Conclusion: Targeting c-Kit plus HER1 or AXL/c-Met abrogates IM resistance in GIST.

## INTRODUCTION

Gastrointestinal stromal tumors (GIST) are defined by mutated and over-expressed oncogenic receptor tyrosine kinases (RTKs) c-Kit or α-PDGFR that are also effective therapeutic targets for which imatinib mesylate [IM (gleevec), Novartis Pharmaceuticals] an ATP-site small molecule tyrosine kinase inhibitor (TKI) is approved as frontline therapy in the advanced [1-4] and adjuvant [5] settings. C-Kit directed therapies have changed the natural history of aggressive GIST and clearly improved survival in both settings [4, 5]. For patients with advanced disease who are IM resistant or intolerant, sunitinib malate (SM), a c-Kit/PDGFR/VEGFR TKI is approved as second line therapy [6] and regorafenib in the third line setting [7] for those patients progressed on IM and SM. Oncogenic c-Kit mutations (~85%) in GIST result in constitutive RTK activation: the most common exon 11 juxtamembrane domain mutations (~70%) (proximal and distal) [8] are IM sensitive [3]. However, patients failing IM with exon 11 mutations acquire secondary mutations within the ATP-binding site or activation loop and are likely also to be resistant to SM [9]. Extracellular domain exon 9 c-KIT mutations (~12%) are frequently associated with small intestinal GIST and require higher doses of IM for response (600 or 800 mg) but this mutation appears also to be sensitive to SM [2, 4]. Exon 13 N-terminal kinase domain mutations (~1%) are rare and IM sensitive, while exon 17 activation loop mutations, also rare (~1%) are IM resistant [4, 8]. Approximately 10% GIST is wild type c-Kit and resistant to IM but sensitive to SM [8, 10]. About 5-7% of GISTs have juxtamembrane (exon 12) or activation loop (exon 18) mutations in the α-PDGFR that are mutually exclusive with wild type c-Kit [11] α-PDGFR mutants are generally IM resistant but may be SM sensitive [12].

Chronic c-Kit directed therapies with IM or SM lead to emergence of drug resistant GIST in >50% [~6 months to 3 years] and there is a clear need for a better biologic understanding of the genetic mechanisms of evolution of drug resistance. Several alternative potential mechanisms of drug resistance to IM and SM are currently under active investigation. These include hemi- or homozygous deletion of the wild type *Kit* allele [13], BRAF V600E mutation (5% GIST) [14], a RTK switch (loss of c-Kit and gain of AXL) [1], over-expression of focal adhesion kinase (FAK) [15] and insulin like growth factor receptor I (IGF-1R) amplification [16]. For patients who fail both IM and SM and continue to have a good performance status, an appropriate clinical trial is recommended [17]. However, the development of novel targeted agents and their rational combinations are urgently required to prevent and treat IM or SM resistance.

Immunohistochemistry (IHC) analysis of several oncogenic RTKs in GIST patient specimens demonstrated uniform expression of c-Kit and HER-1, while IM resistant patients express IGF-1R and AXL. In 3 GIST cell lines with single (GIST882) and double (GIST430/654 and GIST48) c-Kit mutations, c-Kit is over-expressed in comparison to HER1 and c-Met expression which corroborates with patient samples. Acute treatment of GIST882 cells with IM leads to up-regulation of c-Kit expression, while chronic IM treatment leads to loss of c-Kit expression. The differential sensitivity of the GIST cell lines targeting c-Kit plus HER1 or c-Kit plus AXL/Met provide a rationale to abrogate resistance that develops with acute and chronic IM therapy in GIST.

## RESULTS

### GIST Patient Characteristics

Sixteen patient cases were divided into two cohorts A and B (Table 2). In Cohort A, two samples were analyzed for Patients 2 and 4 and for Patient 1 there were three. These samples corresponded to separate surgical resections over the span of several years. Tumor samples from six patients (1, 2, 4, 6, 7, and 8) were resected prior to IM treatment and five samples (1, 2, 3, 4, and 5) were post-IM surgical specimens. Patients (1, 2 and 4) had both pre- and post- IM samples. There were 8 males (53%), 4 females (27%), and 3 of unknown gender. The mean age for all samples was 58 years (51-93 years). There were 7 Caucasians (47%), 1 Asian (0.1%), 2 Hispanics (13%), and 5 of unknown ethnicity (33%). An additional patient (patient 16) (Table S1) was included for Western blotting analysis for c-Kit expression.

**Table 1:** Antibodies for IHC Analysis All targets for IHC analysis including species and vendor

Antibody Target	Antibody Vendor	Catalog No.	Species	Clonality	Clone No.
c-Kit (CD117)	Ventana	790-2951	Rabbit	Monoclonal	9.7
Axl	Sigma	WH0000558M1	Mouse	Monoclonal	6C8
c-Met	Ventana	790-4430	Rabbit	Monoclonal	SP44
EGFR	Ventana	790-4347	Rabbit	Monoclonal	5B7
Her2/Neu	Ventana	790-100	Rabbit	Monoclonal	4B5
IGF-1R	Ventana	790-4346	Rabbit	Monoclonal	G11
EGFR (L858R Mutant Specific)	Cell Signaling Technology	3197	Rabbit	Monoclonal	43B2
EGFR (E756-A750 Deletion Specific)	Cell Signaling Technology	2085	Rabbit	Monoclonal	6B6
PTEN	Cell Signaling Technology	9188	Rabbit	Monoclonal	D4.3

**Table 2:** GIST Patient Demographics Demographics for 15 of the 16 patients were recorded

Cohort	Patient	Sex	Ethnicity	Age	Tumor Location	Pre/Post–Surgery IM
A	1	Female	Asian	51	omentum	pre
				53	omentum	post
				54	cecum	post
	2	Male	Caucasian	73	small bowel	pre
				76	omentum	post
	3	Male	Hispanic	48	small bowel	post
	4	Male	Caucasian	64	small bowel	pre
				66	colon	post
	5	Male	Caucasian	53	pelvis	post
	6	Male	Caucasian	63	stomach	pre
	7	Female	Hispanic	67	stomach	pre
	8	Male	Caucasian	90	stomach	pre
B	9	Female	Caucasian	unknown	stomach	pre*
	10	Female	Caucasian	unknown	unknown	pre*
	11	unknown	unknown	unknown	small bowel	pre*
	12	Male	unknown	83	gastric	pre*
	13	Male	unknown	93	gastric	pre*
	14	unknown	unknown	unknown	unknown	pre*
	15	unknown	unknown	unknown	unknown	pre*

* Not confirmed; assumption made based upon rarity and source of samples

### RTK Biomarker Panel Characterization

**Figure 1:**
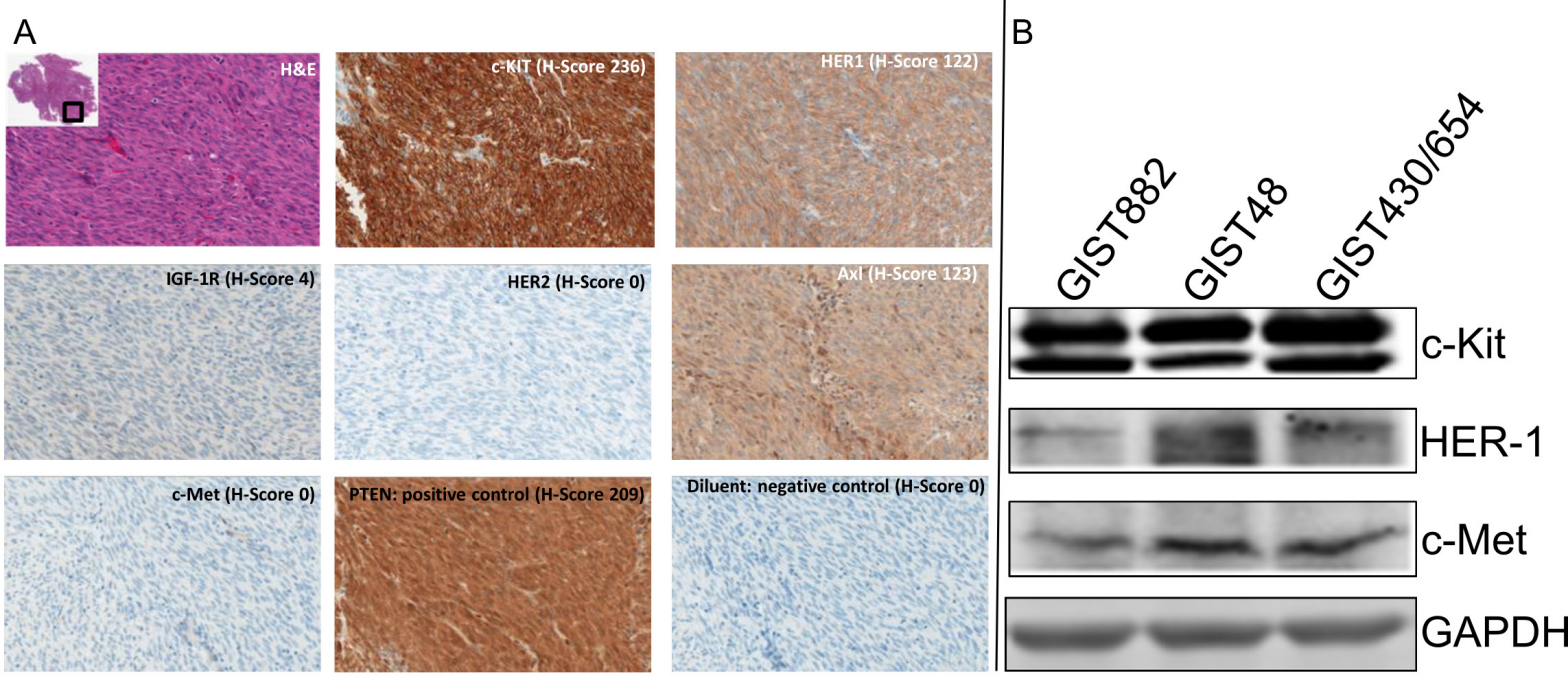
Immunohistochemistry Analysis (A). Immunohistochemistry Analysis of Receptor Tyrosine Kinases (c-Kit, HER-1, IGF-1R, HER-2, AXL and c-Met) with H/E staining, PTEN (positive control) and diluent (negative control), in GIST Specimens. Representative photomicrographs of patient 1 with H-Score (scale 0-300) and magnification = 20x. (B). Western blotting analysis for c-Kit, HER-1 and c-Met expression in GIST882, GIST48 and GIST430/654 cells.

A panel of 6 receptor tyrosine kinases (RTKs) by IHC assays was used to characterize 15 GIST samples. Representative images of patient 1 are shown in Figure 1A. Positivity across all samples was defined as the tumor displaying at least 10% of tumor cells staining (Table 3). An H-score was used to assess staining intensity (Table S2). As expected, c-Kit expression was seen in 14 of 15 tumors (93%) with a mean intensity of an H-score of 165 (range of 0-259). Protein expression was observed for the other RTKs: HER1 - 14/15 (93%), mean H-score of 73 (range 0-179); IGF-1R - 3/15 (20%), mean H-score 93 (range 0-137); AXL - 15/15 (100%), mean H-score of 111 (range 14-220). All samples were negative for c-Met and HER-2. One patient (9) had negative staining across all markers except for low AXL staining.

Across all samples, HER-1 staining was lower than c-Kit. No differences were observed in the expression levels of c-KIT, HER-1 or PTEN when samples were grouped based upon sex, pre/post IM, or cohort when data were analyzed by t-Test (Table 4). PTEN was used to show that any potential differences seen were not due to pre-analytical parameters.

Western blotting of GIST882, GIST48 and GIST430/654 cells indicated all 3 cell lines express c-Kit, HER1 and c-Met but the level of expression is 10-20 fold higher for c-Kit compared to HER1 and c-Met (Figure 1B). GIST cell line data correlate well with IHC results for RTK expression from debulked tumors.

**Table 3:** Cells/Pixels Staining Positive Percent of cells/pixels staining positive for various tested antibodies in GIST patient samples

Cohort	Patient	Pre/Post Surgery IM	c-Kit (%)	HER1(%)	IGF-1R (%)	c-Met (%)	HER2 (%)	Axl(%)	(−) Control(%)	(+) Control (%) (PTEN)
A	1	Pre (A)	85	65	0	0	0	65	0	85
		Post (B)	48	38	0	0	0	11	0	94
		Post (C)	94	69	3	0	0	76	0	99
	2	Pre (A)	77	63	3	2	1	60	0	90
		Post (B)	18	37	0	0	0	52	0	77
	3	Post (A)	93	0	1	1	0	75	0	97
	4	Pre (A)	70	38	70	0	0	45	0	57
		Post (B)	72	48	57	2	2	85	1	90
	5	Post	0	29	56	2	1	46	0	74
	6	Pre	24	17	1	1	1	52	0	89
	7	Pre	90	18	0	0	0	48	0	96
	8	Pre	91	74	2	1	0	88	0	96
B	9	Pre*	0	0	0	0	0	16	0	74
	10	Pre*	57	19	3	2	1	98	0	96
	11	Pre*	90	67	2	1	0	88	0	94
	12	Pre*	86	62	3	2	1	88	0	86
	13	Pre*	92	52	4	1	1	97	0	94
	14	Pre*	91	86	11	7	3	98	2	99
	15	Pre*	96	36	7	2	1	89	1	99

* Not confirmed; assumption made based upon rarity and source of samples

**Table 4:** t-Test The t-Test for two-sample assuming unequal variances for c-Kit, HER-1 and PTEN. Across all samples, EGFR staining was lower than c-Kit. No differences were observed in the expression levels of c-KIT, EGFR, or PTEN when samples are grouped based upon sex, pre- and post-IM, or cohort when data were analyzed by t-Test. PTEN was used to show that any potential differences seen were not due to pre-analytical parameters.

Category	p-value	p-value	p-value
Test	c-Kit	HER1	PTEN
Male vs Female	0.71	0.60	0.39
Coort 1 vs 2	0.40	0.71	0.14
Pre vs Post IM	0.36	0.78	0.12

### HER-1 and IGF-1R *In-Situ* Hybridization

Fifteen GIST samples were analyzed for presence of HER1 and IGF-1R gene amplification by Silver In-Situ Hybridization (SISH). All samples analyzed for both probes contained normal gene copy numbers (~2 copies) signifying that the *HER-1* and the *IGF-1R* genes were not amplified.

### HER-1 mutation and deletion

IHC assays detecting the L858R mutant and E746-A750 deletion of HER-1 were performed on all samples. Using the previously defined criterion of IHC staining of 10% of tumor cells or greater, all samples were negative for both the point mutation and frame shift deletion. Of note however, one sample (14) had faint blush amounts of staining present for the E746-A750 deletion assay, however this was below threshold values set for this assay.

### IM resistant GIST patients demonstrate loss of c-Kit, gain of c-Met and AXL

**Figure 2:**
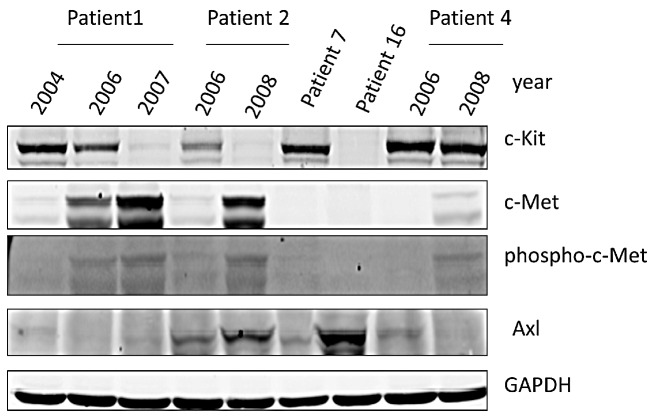
Western Blotting for c-Kit, c-Met, phosphor-c-Met, and AXL in GIST Patient Samples GIST patients [1, 2, 4, 7 and 16] analyzed by Western blotting for c-Kit, c-Met, Phospho-c-Met and AXL expression. Patient 1, 2 and 4 had serial specimen's available pre- and post-IM. A piece of frozen tissue was homogenized, lysed with NP-40 lysis buffer and 50 μg total protein was resolved by electrophoresis on a 10% SDS-PAGE. Immunoblotting was performed using anti-c-Kit, anti-c-Met, anti-phospho-c-Met and anti-AXL antibodies, respectively. GAPDH is used as a loading control.

In order to ascertain whether the cell culture model recapitulates [1] the human situation, we investigated 5 GIST patients that had progressed on chronic IM therapy and had debulking surgeries as part of their management strategy (Table S1). Based on an expert pathology review, snap frozen active tumors were analyzed by Western blotting for expression of c-Kit, c-Met and AXL along with phosphorylation of c-Met (Figure 2). Patient 1 (c-Kit+) progressed on IM and the biopsy at debulking surgery continued to be c-Kit positive. Patient 1 was treated with AMG706 (a c-Kit/VEGFR SMI, Phase II clinical trial) but continued to have progressive disease. On subsequent biopsy at the second debulking surgery (2007), c-Kit expression was negative by IHC. Similarly, patient 2 (c-Kit+) treated with IM for 2 years had progressive disease and IM dose was increased from 400 mg to 600 mg with stabilization of disease. Due to progressive disease, a biopsy from a debulking surgery showed continued c-Kit positivity. Patient 2 then was started on SM with disease stabilization. However, due to progressive symptomatic disease, another debulking surgical biopsy showed c-Kit negativity by IHC. Patient 7, c-Kit maintains over-expression on IM therapy with no evidence of AXL or c-Met induction. However, patient 7 was resistant to IM and SM, which may be due to acquired secondary mutations. Patient 16, c-Kit expression is lost with IM treatment but there is induction of AXL expression. Patient 4, c-Kit continues to be over-expressed despite IM/SM therapy and there is a modest expression of AXL and c-Met RTKs. The resistance to IM here appears to be mixed (secondary c-Kit mutations and alternative RTK expression). Together these observations support the notion that RTK driven down stream signaling pathways continue to be activated and provide a survival and proliferative advantage in c-Kit negative GIST patients.

### Development of IM resistance with short and long term culture of GIST cells

The IC_50_ for IM in the GIST882 cell line is 0.077 – 0.14 μM [1]. When confluent GIST882 cells are incubated in IM at 0.5 μM (3.5 – 6.5 fold x IC_50_), they can be cultured with continuous exposure to IM. GIST882 cells were treated with 0.5 μM IM for 1, 2, 4 weeks (acute) and 2, 3, 4 and 6 months (chronic) maintained to confluence. In short term culture, RT-PCR for c-Kit showed a 1.5-fold decrease in mRNA compared to untreated cells with duration of treatment (Table 5). In contrast, c-Met and AXL message RNA increased by ~3-fold and ~2.4-fold respectively compared to untreated cells. Furthermore, HGF (c-Met ligand) and Gas6 (AXL ligand) message RNA increased ~8-fold and ~20-fold respectively implicating an autocrine mechanism of activation. These results suggest that acute therapy with IM may lead to loss of c-Kit expression with gain of expression and activation of AXL and c-Met via an autocrine loop.

**Figure 3:**
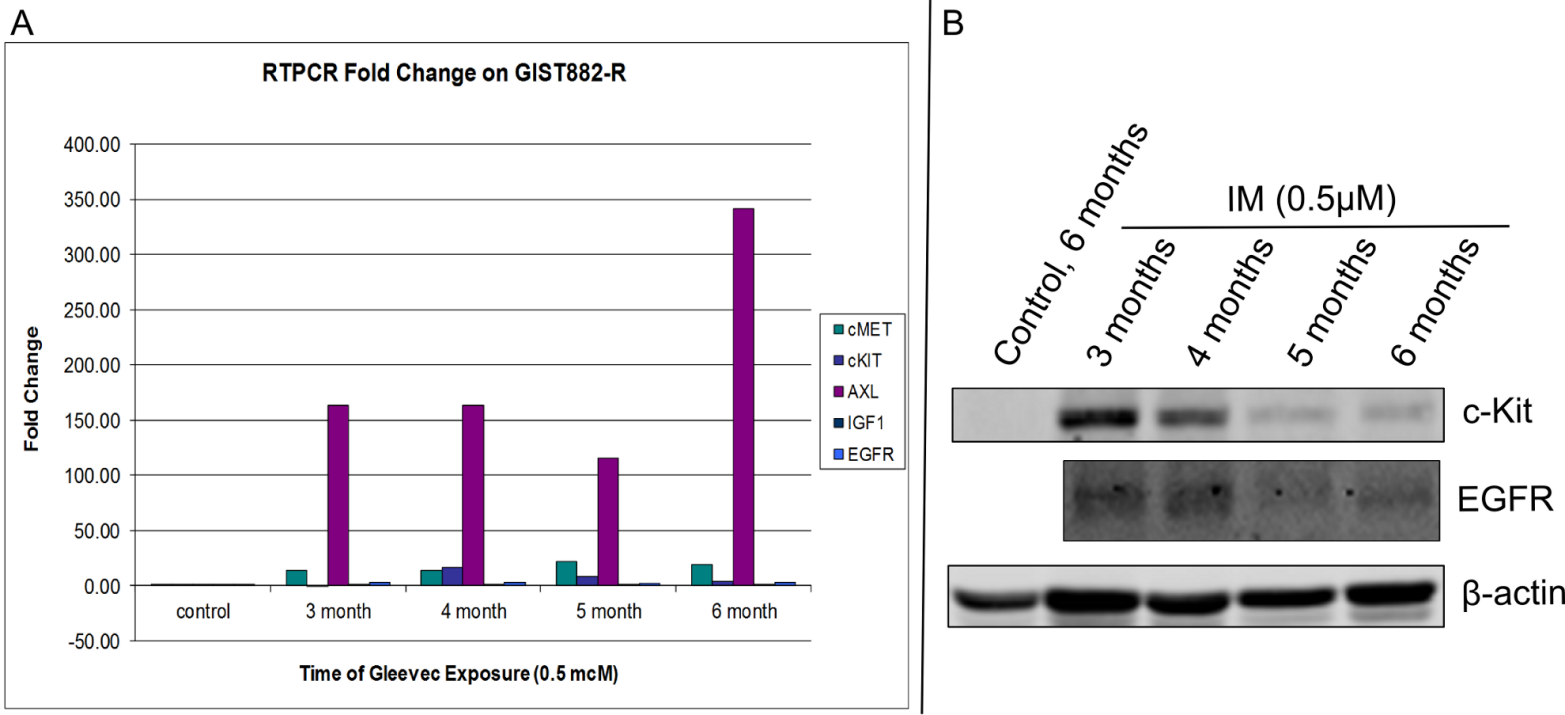
Quantitative RT-PCR of GIST 882-R Cells (A). Quantitative RT-PCR of Receptor Tyrosine Kinases (c-Kit, c-Met, AXL, IGF-1R and HER-1) after treatment of GIST882 cells with IM (0.5μM) every other day for 3, 4, 5 and 6 months and (B). Western Blotting for c-Kit and EGFR down-regulation measured at 3, 4, 5 and 6 months in the presence of IM (0.5μM). Beta-actin protein was used as a loading control.

In medium to long term culture of GIST882 cells treated with IM (0.5μM), showed a significant decrease in the c-Kit message from 27-fold at 3 months to 4-fold at 6 months compared to control (Figure 3A). This observation was confirmed by c-Kit protein level which decreased by ~50% at 4 months (compared to 3 months) and complete loss at 5 and 6 months (Figure 3B) consistent with our prior observations [1]. HER1 expression also diminished at 4 months of IM therapy (Figure 3B). The ‘control, 6 months’ (Figure 3B) are GIST882 cells grown without IM with the media changed every other day. The GIST882 cells were not split during the course of the study in order to maintain continuity between the samples and reached 100% confluency prior to the 6 month termination of the experiment. These GIST cells do not express c-Kit, most likely, due to cell cycle arrest. RT-PCR for AXL showed dramatic increases in message from 120 to 340-fold compared to control (Figure 3A). However, c-Met showed an increase message from 14-fold (3, 4 months) to 18 to 22-fold (5, 6 months) compared to control (Figure 3A). In contrast, no changes in mRNA levels were observed for IGF-1R and HER-1 with duration of treatment (Figure 3A).

**Table 5:** Quantitative RT-PCR after Acute Treatment of GIST 882 Quantitative RT-PCR of Ligand (HGF, GAS6) and Receptor Tyrosine Kinases (c Kit, c-Met and AXL) after treatment of GIST882 cells with IM (0.5μM) for 1, 2 and 4 weeks.

		Fold change ± 95% CI for given genes			
Treatment Duration	HGF	c-Met	GAS6	Axl	c-Kit
1 week	7.90±0.20	2.74±1.20	20.63±1.26	1.02±1.09	1.20±0.90
2 weeks	7.40±0.37	2.81±1.52	17.98±1.11	2.35±1.46	−1.5±0.79
4 weeks	8.90±0.36	4.67±1.34	23.99±1.17	2.02±1.18	−1.3±0.78

### Novel RTK combination therapies overcome IM-resistant GIST

**Figure 4:**
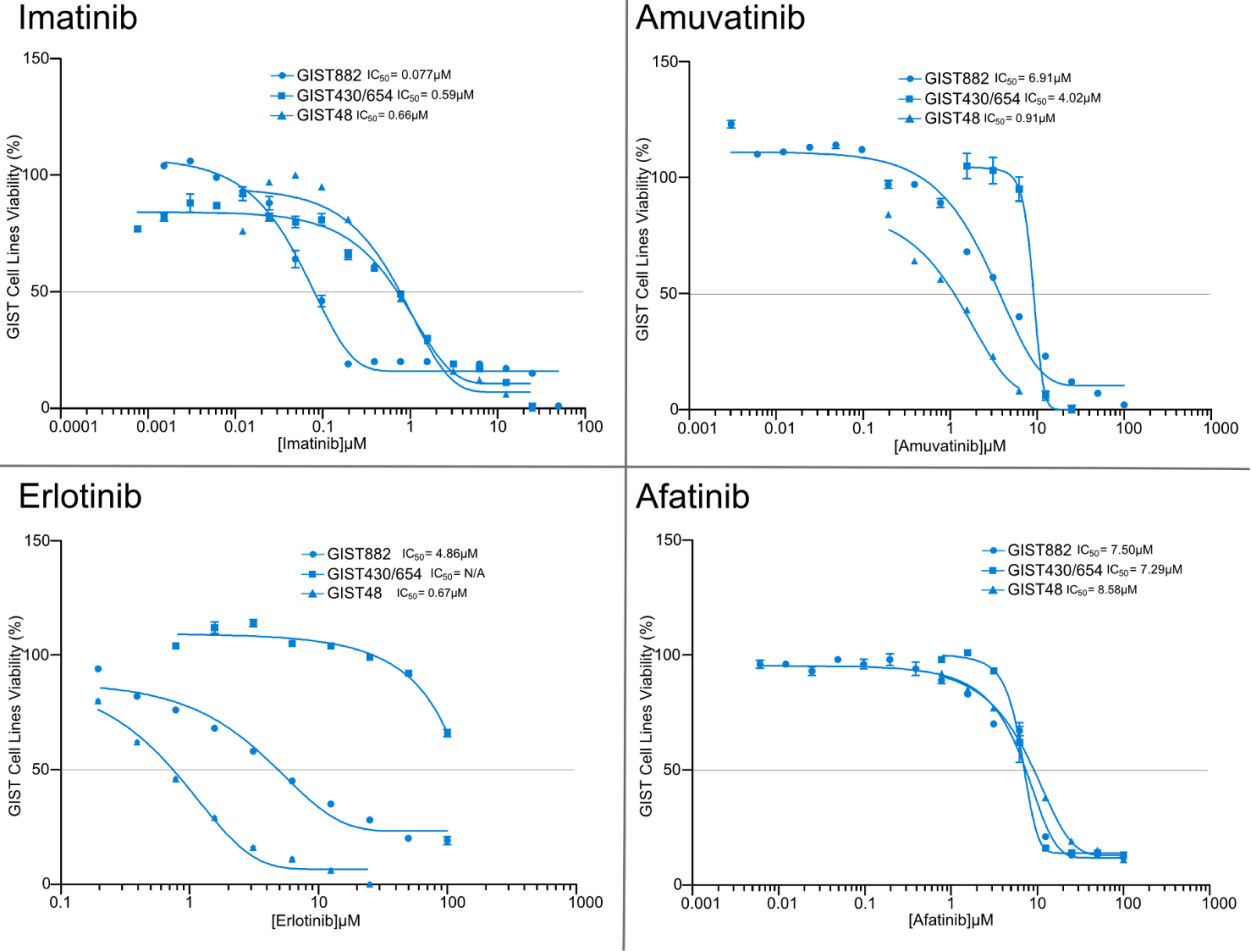
MTS Cytotoxicity Activity of Single Agents MTS cytotoxicity activity of single agents IM, amuvatinib, erlotinib and afatinib represented as IC_50_ (μM) in GIST882, GIST48 and GIST430/654 cells.

The goal was to investigate the efficacy of single agent RTK SMIs (IM, SM, amuvatinib, erlotinib, afatinib) on IM sensitive and resistant GIST cell lines. MTS cytotoxicity assays for GIST882, GIST48 and GIST430/654 cells treated with single agent serial dilutions of IM, amuvatinib, erlotinib and afatinib with IC_50_ (μM ±95% CI) are shown in Figure 4 and tabulated (Table 6) indicated differential sensitivity and resistance to therapy. Included in Table 6 are the IC_50_ data for SM. GIST882 cells are sensitive to IM (0.077 μM) and SM (0.068 μM) but resistant to amuvatinib (6.91 μM), erlotinib (4.86 μM) and afatinib (7.50 μM) (Table 6); GIST48 cells are sensitive to IM (0.66 μM), amuvatinib (0.91 μM) and erlotinib (0.67 μM) but resistant to sunitinib (4.65 μM) and afatinib (8.58 μM) (Table 6); GIST430/654 cells are sensitive to SM (0.15μM) and IM (0.59 μM) and resistant to amuvatinib (4.02 μM) and HER1 inhibitors (Table 6).

**Figure 5:**
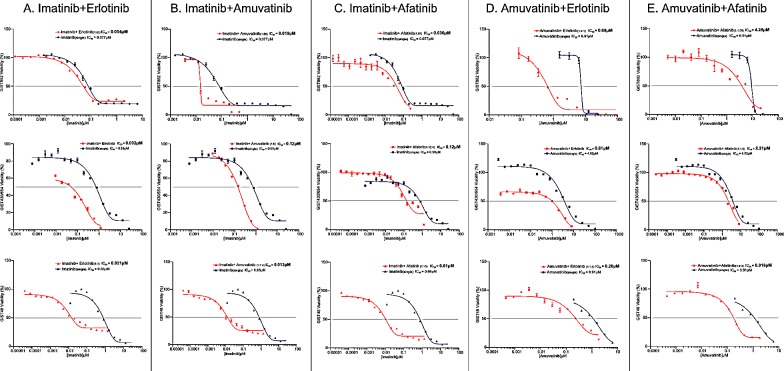
Combination Therapy The relationship of combinations with IM (A-C) or amuvatinib (D and E) with erlotinib or afatinib determined by calculating the ratios of their respective ED_50_ values from the single dose studies.

Combination studies were conducted to determine the equipotent ratios for the 3 GIST cell lines. The relationship of combinations with IM (Figure 5A,B,C) and amuvatinib (Figure 5D, E) were determined by calculating the ratios of their respective ED_50_ values from the single dose studies. Equipotent ratios for the respective combinations are incorporated within Figure 5A-E. Combinations (CI values) with IM show strong synergy with HER1 SMIs in GIST48 and GIST430/654 cells. In contrast, amuvatinib plus HER1 SMIs are strongly synergistic in all 3 GIST cell lines. An antagonistic pharmacological interaction is observed in GIST882 cells with IM plus erlotinib and amuvatinib plus afatinib (Table 7). Moreover, amuvatinib plus afatinib is also antagonistic in GIST430/654 cells.

**Figure 6:**
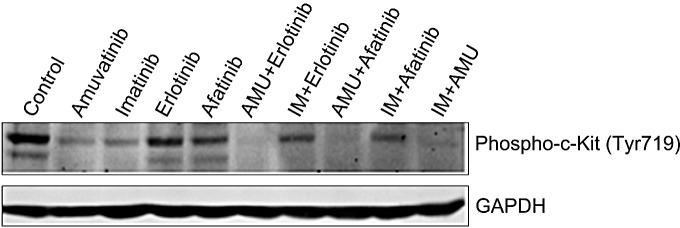
GIST48 Cells Treated with Synergistic Combinations of RTK Inhibitors GIST48 cells were treated with IM (0.6μM), amuvatinib (AMU, 1.0μM), erlotinib (0.7μM), afatinib (8.5μM), AMU (1.0μM) + erlotinib (0.7μM), IM (0.6μM) + erlotinib (0.7μM), AMU (1.0μM) + afatinib (8.5μM), IM (0.6μM) + afatinib (8.5μM) and AMU (1.0μM) + IM (0.6μM) for 1h. Western blotting was performed for c-Kit phosphorylation (Y719). GAPDH was used as loading control.

Western blotting analyses of GIST48 cells treated with synergistic combinations amuvatinib + erlotinib, or amuvatinib + afatinib or amuvatinib + IM at IC_50_ at 60 min indicate complete inhibition of c-Kit phosphorylation (Y719) in comparison to single agent IM, amuvatinib, erlotinin and afatinib (Figure 6). MTS cytotoxicity corroborates with *in vitro* phosphorylation of the c-Kit enzyme activity as a mechanism of efficacy for combination therapy.

**Table 6:** Single Agent IC_50_ Values ± 95% Confidence Interval Single agent activity represented as IC_50_ (μM ± 95% CI) for IM, SM, amuvatinib, erlotinib and afatinib in GIST882, GIST430/654 and GIST48 cells.

Drug	GIST882	GIST48	GIST430/654
**Imatinib**	0.077±0.01	0.66±0.38	0.59±0.49
**Amuvatinib**	6.91±1.81	0.91±0.62	4.02±1.30
**Erlotinib**	4.86±1.50	0.67±0.38	N/A
**Afatinib**	7.50±1.86	8.58±1.69	7.29±0.81
**Sunitinib**	0.068±0.003	4.65±0.25	0.15±0.05

**Table 7:** Combination Index Values ± Standard Deviation Combination-Index ± SD for IM plus amuvatinib or afatinib or erlotinib in GIST882, GIST430/654 and GIST48 cells.

Cell Line	GIST882		GIST48		GIST430/654	
Drug	Imatinib	Amuvatinib	Imatinib	Amuvatinib	Imatinib	Amuvatinib
**Erlotinib**	1.42±0.50	0.201±0.06	0.63±0.0075	0.823±0.070	N/A	N/A
**Afatinib**	0.93±0.33	1.24±0.27	0.031±0.0018	0.362±0.030	0.397±0.043	1.10±0.23
**Imatinib**	-	0.50±0.15	-	0.041±0.0066	-	0.42±0.051

## DISCUSSION

In GIST patients, drug resistance is associated with distinct molecular and clinical features due to development of secondary mutations within the c-Kit kinase domain and alternative mechanisms of resistance [1, 9, 10]. However, with acute IM therapy c-Kit+ GIST cells undergo apoptosis via histone H2AX, but a side population of cells enters G0 and quiescence [19]. With acute-on-chronic IM therapy, c-Kit is down-regulated and new RTKs (e.g. c-Met, AXL) gain expression in a novel ‘RTK switch’ [1]. The underlying questions regarding the specific cause(s) of drug resistance, how tumors adapt, and new treatment options required to abrogate drug resistance needs further investigation.

We examined the expression patterns of several RTKs (c-Kit, c-Met, AXL, HER-1, HER-2, and IGF-1R) in GIST patients by IHC to identify therapeutic opportunities that may help prevent and/or overcome drug resistance. Expression of c-Kit in 14 of 15 tumors (93%) was strong and homogeneous confirming signaling via this RTK is a primary driver of tumor growth [20]. Further, HER-1 was also homogenously positive in 14 of 15 GIST specimens (93%) [21], with patient 9 lacking both c-Kit and HER-1. A few GIST cases do have *HER-1* amplification (5.3%) and HER1 expression was reported in 96% of GIST samples tested [22]. No evidence of HER1 gene amplification was detected in 15 GIST samples consistent with a FISH analysis of GIST [23]. We did not detect the existence of HER-1 E746-A750 deletion and L858R point mutation [24], where 60 GIST were sequenced for *HER-1* demonstrated no mutations.

IGF-1R was positive only in patients 4 and 5 with a normal *IGF-1R* gene copy number indicating it is a rare RTK expressed on GIST. Patient 4 had medium to strong expression for c-KIT, HER-1, and IGF-1R. In contrast, patient 5 was c-Kit negative and weakly positive for HER-1. Fifteen GIST specimens tested by IHC for c-Met and HER-2 were negative but all stained positive for AXL (Figure 1A). Western blotting analysis of GIST patients showed AXL positivity in 1, 2, 4, 7 and 16 and c-Met positivity in 1, 2 and 4 (Figure 2, Table S1).

The development of IM resistance in short-term culture of GIST882 cells treated with a high dose of IM (3.5-fold dose of IC_50_) for 1, 2 and 4 weeks demonstrated c-Kit over-expression at 1 week, followed by loss of expression at 2 and 4 weeks (Table 5). In contrast, ligand-receptor pairs HGF-c-Met and Gas6-AXL are over-expressed in a time-dependent manner (Table 5). In 2 IM-resistant patients, [1], RT-PCR for HGF and Gas6 showed increased expression and both of these patients also over-expressed c-Met and AXL (Figure 2) implying an autocrine mechanism of activation. In GIST-T1 cells (*c-Kit* exon 11 mutation) over-expression of Cas-L and Src activation was proposed to be a mechanism of resistance to IM [25] but no confirmatory data was provided in human GIST specimens.

Since several RTKs are expressed in GIST patients (Figure 1A) and GIST cell lines (Figure 1B), we evaluated single agent activity (Figure 4, Table 6) and combination therapy (Figure 5, Table 7) for synergy that may help overcome drug resistance. Of the single agents, IM is 10-fold more active in GIST882 (exon 11 K642E) than in GIST48 (exon 11 and 17 missense mutation) or GIST430/654 (exon 11 in-frame deletion and exon 13 V654A) cells. SM is equipotent to IM in GIST882, however, is more potent that IM in GIST430/654 but inferior to IM in GIST48 cells likely due to the c-Kit exon 17 mutation. Amuvatinib and erlotinib are equally active in GIST48 cells but have modest activity in GIST882 and GIST430/654 cells (Table 6) most likely due higher expression of c-Met and HER1 respectively (Figure 1B). In GIST882 cells, IM plus erlotinib was antagonistic, however, in GIST430/654 and GIST48 cells this combination showed strong synergy and corroborated with that observed for IM plus afatinib. The ineffectiveness of IM plus erlotinib or afatinib in GIST882 cells is likely due to activation of alternative signaling pathway(s). Highly synergistic combinations for GIST882 are AMU + erlotinib (CI 0.20) and IM + AMU (CI 0.50); for GIST430/654 are IM + afatinib (CI 0.39) and IM + AMU (CI 0.42); for GIST48 are IM + afatinib (CI 0.03), IM + AMU (CI 0.04), AMU + afatinib (CI 0.36) and IM + erlotinib (CI 0.63) (Table 7). GIST48 cells treated with synergistic combinations of RTK inhibitors at IC_50_ demonstrated complete inhibition of c-Kit phosphorylation (Y719) in comparison to single agent IM (Figure 6) implicating cross-talk between these pathways. Signaling through c-Kit remains pivotal to all 3 GIST cell lines, however, inhibition of c-Met and HER1 provides an additional mechanism to turn off dominant c-Kit signaling. The mechanism of pharmacologic antagonism of IM plus erlotinib and amuvatinib plus afatinib in GIST882 and GIST48 cells in under investigation.

Collectively, our findings suggest that targeting multiple RTKs on GIST is more effective than single agent RTK therapy targeting predominantly c-Kit or α-PDGFR and/or VEGFR2. Given a broader knowledge of the mutational status and expression levels of c-Kit in IM-sensitive and resistant GIST patients, targeting c-Kit plus HER1 (IM or amuvatinib plus erlotinib or afatinib) or c-Kit plus c-Met/AXL (IM plus amuvatinib) with novel combinations of RTK inhibitors abrogates IM resistance in GIST. Synergistic relationships between drugs are the most advantageous interactions in terms of response rates and reduced side effects and should be pursued as potential novel therapeutic strategies for treatment of GIST. Clinical trials are warranted to evaluate these combinations in GIST patients failing c-Kit directed therapy.

## MATERIALS AND METHODS

### GIST Patient Specimens

Through an IRB approved protocol, IM and/or SM resistant GIST patients who had failed [tumor(s) had grown >20% from baseline using Response Evaluation Criteria for Solid Tumors] after >2 months of treatment with IM at 600 mg or 800 mg or SM at 50 mg (4 weeks on/2 weeks off) or 37.5 mg orally daily respectively were banked as both frozen and paraffin samples. All tissue samples were de-identified in accordance with HIPPA regulations. There were also five GIST patients who underwent debulking surgery at various times for symptomatic progressive disease and excess tissue was divided for paraffin and frozen sections. All patient materials were supplied as formalin fixed, paraffin embedded (FFPE) tissues. Two cohorts of patient samples were obtained to investigate receptor tyrosine kinase expression. Tissue blocks and patient information of Cohort A (n=9) were obtained from Dr. Mahadevan's GIST patients (pre- and post-IM) from the University of Arizona Cancer Center (Tucson). Cohort B samples (n=7) were GIST patients (IM naïve) de-bulked prior to IM therapy obtained from Ventana Medical Systems (Tucson).

### GIST IHC and Western Blotting

Immunohistochemistry (IHC) was performed using Ventana's BenchMark XT automated staining platform in collaboration with Arizona Cancer Center (AZCC) tissue acquisition and cellular/molecular analysis (TACMASS, Dr. R. Nagle). Antibodies to c-Kit, AXL, c-Met, HER1, HER2, HER1 (L858R mutant specific), HER1 (E746-A750, deletion specific), IGF-1R, and PTEN were utilized for IHC analysis (Table 1). The FFPE tissue samples were sectioned at 4 μM and transferred in a water bath onto Superfrost Plus slides (VWR, Catalog No. 48311-703). H&E stains were performed on all tissues prior to IHC. The H&E stains were reviewed by a pathologist (Dr. R. Nagle) to verify tumor presence, tissue integrity and cell viability. Staining for PTEN was used as a positive control to evaluate tissue quality. Samples are flagged if PTEN staining of these elements was weak or uneven across the specimen and rejected if there was no staining. Antibody diluent was substituted for the primary antibody as a negative control across all samples. All IHC assays used the *ultra*View Universal DAB detection chemistry (Ventana; Cat #760-500). The secondary antibody, horseradish peroxidase (HRP) is a biotin-free multimer complex. For these assays, the secondary antibody and all chromogen reagents were applied using the instrument's default times. Snap-frozen sections were utilized for Western blotting analysis for c-Kit, c-Met and AXL expression and c-Met phosphorylation.

### *In-Situ* Hybridization

Ventana's fully automated silver *in situ* hybridization method (SISH) was developed to detect the HER1 gene status in FFPE tissues (VMSI, Catalog No. 780-001). Staining was performed on Ventana's BenchMark XT automated slide platform. In the present study, scoring consisted of distinguishing non-amplified (~2 gene copies) from amplified (>2 gene copies) status.

### Reagents and Cells

Imatinib mesylate (Gleevec, Novartis Pharmaceuticals) was purchased from 21CEC PX Pharmaceuticals Ltd (Shanghai, China). Sunitinib malate (Sutent, Pfizer Pharmaceuticals) was received from Pfizer oncology (investigator initiated research grant to Dr. Mahadevan). Amuvatinib (MP470) and erlotinib (150 mg tablets, Genentech, CA) was synthesized and purified respectively by the University of Arizona Cancer Center chemistry core facility (Dr. E. Mash). Afatanib was purchased from Selleck chemicals (Houston, USA). RPMI 1640 tissue culture medium and fetal bovine serum were purchased from Cellgro (Manassas, VA). Anti-c-Kit (C-19) antibody was obtained from Santa Cruz Biotechnology (Santa Cruz, CA). Anti-phospho-c-Kit (Tyr719) and anti-GAPDH (14C10) antibodies were from Cell Signaling Technology (Danvers, MA). Mouse anti-c-Met (3D4) antibody was bought from Zymed (South San Francisco, CA). Anti-Phospho-c-Met (Tyr1230/1234/1235) was from Thermo Scientific (Rockford, IL). Anti-AXL was from Abnova (Walnut, CA) and R&D Systems (Minneapolis, MN), respectively.

IM sensitive (GIST882-S) and IM resistant (GIST882-R, GIST430/654, GIST48) cell lines were cultured in 75 cm^2^ tissue culture flasks with RPMI1640 containing 15% fetal bovine serum and 0.5% L-glutamine. Culture media was replaced three times per week. Cells were grown in a humid atmosphere set at 37^o^C with 5% CO_2_ and were demonstrated to be free of mycoplasma using PCR analysis.

### Cell Viability Assays

GIST882, GIST430/654 and GIST48 cells were seeded into 96-well plates at a cellular density of 5.0 × 10^4^ per well. Cells were incubated for 24 hr to allow for ample time for attachment to the well. After 24 hr cells were treated with graded concentrations of IM, SM, erlotinib, afatinib and amuvatinib in six replicates in 0.1% DMSO including a DMSO only control. For combination studies, treatments were performed in the same manner. Median effects from each of the single treatments were used to establish an equipotent ratio, a ratio of the respective ED_50_'s. The equipotent ratio was utilized to form a dosing scheme from which new dose median effects could be obtained for further analysis of drug interactions. A mixture of drug combinations up to 10-fold the equipotent ratio was administered to the GIST cells. A control group was established for each drug treatment in six replicates. The cells were exposed to the respective treatments for 96 hr. Cell viability was determined through the CellTiter96 Aqueous non-radioactive cell proliferation assay (MTS). Absorbance readings at 490 nm were analyzed against the control group for each drug treatment to determine cell viability.

The efficacies of various treatments were expressed as the dose median (Dm), also known as the effective dose at 50% (ED_50_). The median effect equation f_a_/f_u_ = (D/D_m_)^m^, where f_a_ and f_u_ represent the fraction of cells affected and unaffected by the dose, D. Cell viability curves for each treatment was obtained using GraphPad prism 2.1 (GraphPad Software, CA) and the median effects and statistical analyses were obtained using Calcusyn software (Biosoft, MO). Statistical analysis of the viability assays was conducted using standard error calculations with a 95% confidence interval. Linear correlation coefficient values (r) were determined for each treatment based off the median-effect plots. Sigmoidicity (m) of the D-R curve was determined for each treatment. The effects of the combined treatments were determined by the combination-index and isobologram methods derived from the median-effect principle of Chou and Talalay [18]. The combination-index (CI) method quantifies a combined drug interaction through the analysis of fractional cell kill derived from enzyme kinetic models that determine additive effects. The Calcusyn software was utilized to calculate the CI values for each drug combination for effective dose at 50% (ED_50_) fractional cell kill.

### IM Resistant GIST cell line analysis and drug combination studies

GIST882 cells were treated with 0.5 μM IM for 1, 2, 4 weeks (acute) and 3, 4, 5 and 6 months (chronic) maintained to confluence. Fresh IM was added every other day and any floating cells were removed. At the end of each treatment time point, cells were harvested for analysis of c-Kit, c-Met, HER1, IGF-1R, AXL, HGF and Gas6 by real-time RT-PCR or Western blotting. GIST430/654 (100nM IM) and GIST48 cell are cultured (above) and analyzed for RTK expression.

### Immunoblotting

GIST cells or excess tissue from patients (snap frozen) were lysed in NP-40 lysis buffer containing 50 mM Tris.HCl (pH 7.4), 0.15 M NaCl, 0.5% NP-40, 1 mM DTT, 50 mM Sodium Fluoride, and 2 μl/ml Protease inhibitor cocktail (Sigma, St. Louis, MO). Protein concentrations were determined using the BioRad protein assay kit (Hercules, CA), and 50 μg of protein were resolved by electrophoresis on a 10% SDS-PAGE gel. The proteins were then transferred onto a nitrocellulose membrane and nonspecific binding was blocked by incubating with 5% nonfat milk in TBST buffer (0.01 M Tris-HCl, 0.15 M NaCl, 0.5% Tween-20, pH 8.0) for 1 hr at room temperature. The membrane was subjected to the indicated antibodies, and the proteins were detected by a LI-COR Odyssey Infrared Imaging System. Western blotting was performed to confirm the real time RT-PCR with the indicated antibodies on the GIST882 cell line treated with IM at 0.5 μM for 1, 2 and 4 weeks.

### Quantitative Real Time RT–PCR

Quantitative real-time RT–PCR was carried out on RNAs isolated from GIST cells treated with IM at 0.5 μM for 1, 2 or 4 weeks. First strand cDNA was synthesized by reverse transcription using Superscript III first strand synthesis kit (Invitrogen), and then the real-time PCR was performed on the ABI 7000 real time PCR system (Perkin-Elmer, Branchburg, NJ) with SYBR Green PCR Master Mix (Applied Biosystems, Foster City, CA). The primers (forward/reverse) used in RT-PCR were: c-Kit, c-Met, AXL, IGF-1R, HER1, HGF, Gas6 and internal standard GAPDH. Data is provided as a threshold cycle value (Ct) for each sample, which indicates the cycle at which a statistically significant increase in fluorescence is detected. The data are normalized to GAPDH, which serves as an unaffected control gene for each data point and compared with untreated control to determine relative expression ratios. Each measurement was performed in triplicate.

### IHC Data Analysis

High resolution images of the IHC stained samples were captured using Aperio's (Vista, CA) ScanScope XT. For statistical purposes, the staining intensity categories were converted to a semi-quantitative scale of 0 (negative), 1, 2 and 3. The percentage of pixels staining positively at each intensity level was recorded. In order to generate a single parametric value, H-scores were generated for each sample. An H score, which combines the components of staining intensity and the percentage of positive cells on a scale from 0 to 300, is defined as: [1* (percentage of pixels staining at 1)] + [2* (percentage of pixels staining at 2)] + [3* (percentage of pixels staining at 3)] H-Score. Data analysis was performed using the t-Test: two-sample assuming unequal variances (Microsoft Office Excel 2007).

## SUPPLEMENTARY MATERIAL, FIGURES, TABLES


