# Supplementary material for: Novel receptor tyrosine kinase targeted combination therapies for imatinib-resistant gastrointestinal stromal tumors (GIST)
Source: Oncotarget. 2014 Dec 10;6(4):1954–66. doi: 10.18632/oncotarget.3021 (PMC4385828; doi:10.18632/oncotarget.3021)
Supplement: Supplementary file 1 [file oncotarget-06-1954-s001.pdf]

Novel receptor tyrosine kinase targeted combination therapies for imatinib-resistant gastrointestinal stromal tumors (GIST)

Supplementary Material

Table S1: **Processed Biopsies Pre- and/or Post-IM Therapy for Five GIST Patients.**

Processed biopsy information and c-Kit status for patients 1, 2, 4, and 7 with an additional patient (patient 16) included.

Patient	Age/Sex	Initial Diagnosis	Treatments	Resection	c-Kit
1	51/F	10/2000	IM and AMG706 10/4/2004 (Failed IM in 2005)	12/2004<br>12/2006<br>11/2007	(+)<br>(+)<br>(-)
2	73/M	12/2001	IM since 2002 (Failed IM in 2004)	12/2004<br>9/2006<br>4/2008	(+)<br>(+)<br>(-)
4	64M	9/2002	IM and AMG706 (Failed IM in 2006)	5/2006<br>1/2008	(+)<br>(+)
7	67/F	4/2006	Pre-IM	4/2006	(+)
16	91/F	5/2006	Pre-IM	5/2006	(-)

Table S2: **Calculated H-score for GIST Specimens by Receptor Tyrosine Kinase Expression by Immunohistochemistry.**

Cohort	Patient	Pre/Post Surgery IM	c-Kit	HER1	IGF-1R	c-Met	HER2 (%)	Axl (%)	(-) Control (%)	(+) Control (PTEN)
A	1	Pre (A)	170	88	1	1	0	90	0	170
		Post (B)	62	47	0	0	0	14	0	156
		Post (C)	236	122	4	0	0	123	0	209
	2	Pre (A)	153	88	3	2	1	76	0	163
		Post (B)	25	55	1	0	0	79	0	127
	3	Post (A)	207	0	1	1	0	109	0	194
	4	Pre (A)	132	53	137	0	0	61	0	83
		Post (B)	170	86	118	2	3	163	1	174
	5	Post	0	44	105	2	1	66	0	140
	6	Pre	36	22	2	1	1	72	0	171
	7	Pre	181	19	0	1	0	54	0	183
	8	Pre	230	117	2	1	0	142	0	184
B	9	Pre*	0	0	0	0	0	22	0	138
	10	Pre*	77	22	3	3	1	189	0	180
	11	Pre*	205	103	3	1	0	140	0	183
	12	Pre*	206	85	3	2	1	132	0	165
	13	Pre*	224	67	4	1	1	189	0	194
	14	Pre*	226	179	13	9	4	220	3	249
	15	Pre*	259	44	8	2	1	165	1	215

*Not confirmed; assumption made based upon rarity and source of samples
